# Inhibition of human pancreatic cancer cell (MIA PaCa-2) growth by cholera toxin and 8-chloro-cAMP in vitro.

**DOI:** 10.1038/bjc.1993.53

**Published:** 1993-02

**Authors:** E. Ohmura, K. Wakai, O. Isozaki, H. Murakami, N. Onoda, N. Emoto, K. Shizume, T. Tsushima, H. Demura, R. K. Robins

**Affiliations:** Department of Medicine, Tokyo Women's Medical College, Japan.

## Abstract

**Images:**


Br. J. Cancer (1993), 67, 279-283 © Macmillan Press Ltd., 1993

### Inhibition of human pancreatic cancer cell (MIA PaCa-2) growth by cholera toxin and 8-chloro-cAMP *in vitro*

E. Ohmura[1], K. Wakai[2], O. Isozaki[1], H. Murakami[1], N. Onoda[1], N. Emoto[1], K. Shizume[2], T. Tsushima[1], H. Demura[1] & R.K. Robins[3]

[1]*Department of Medicine, Institute of Clinical Endocrinology, Tokyo Women's Medical College, 8-1 Kawada-cho, Shinjuku-ku;* [2]*Research Institute for Growth Sciences, Shinjuku-ku, Tokyo, 162 Japan;* [3]*Department of Pharmacology School of Medicine, University of California, San Diego 11077 North Torrey Pines Road, La Jolla, California 92037, USA.*

**Summary** The effects of cholera toxin (CT) and 8-chloro-cAMP (8-Cl-cAMP) on cell growth were investigated using two human pancreatic carcinoma cell lines (MIA PaCa-2, Panc-1). CT, which catalyses the ADP ribosylation of Gs, suppresses the proliferation of MIA PaCa-2(PC) cells. CT at the low dose of 0.1 pg $ml^{-1}$ was inhibitory of PC cell growth, and the maximum suppression (70%) was achieved at a CT concentration of 100 pg $ml^{-1}$. This phenomenon was reversible. The production of cAMP by CT (100 pg $ml^{-1}$) in PC cells was enhanced 320-fold compared with the control. In addition, cAMP analogues (8-Cl-cAMP, 8-Br-cAMP) and forskolin decreased the growth rate of PC cells in a dose-dependent manner. These results support the view that CT suppresses PC cell growth by stimulating cAMP production. Conversely, Panc-1 cells were far less sensitive to CT in cell growth and cAMP production. 8-Cl-cAMP was also less effective on Panc-1 cell growth.

The binding of an insulin-like growth factor (IGF)-I and transforming growth factor (TGF)-α, which has been shown to stimulate PC cell growth in an autocrine manner, to PC cells was not modified in cells treated with CT or 8-Cl-cAMP. The results suggest that the inhibitory actions of these substances do not occur at the level of the receptor for IGF-I or EGF/TGF-α. We have previously shown that phorbol esters, which decrease the binding of TGF-α to PC cells, has an anti-proliferative activity on these tumour cells. Inhibited cell growth by maximum suppressive dose of CT or 8-Cl-cAMP was further inhibited by TPA. In addition, an oncogene product of K-ras which is commonly activated in pancreatic cancer, was increased by CT and 8-Cl-cAMP.

It is concluded that CT and 8-Cl-cAMP inhibit PC cell growth, presumably in a similar manner, and their mechanism(s) of action may be different from that of TPA. The anti-proliferative effect of CT or 8-Cl-cAMP was enhanced by TPA, implying that the combination of these substances results in increased inhibition of the PC cell growth.

The bacterial exotoxin, cholera toxin (CT), is known to bind with high affinity to monosialoganglioside $G_{M1}$ on the cell surface and stimulate ADP-ribosylation of the stimulating G protein of adenylate cyclase, $G_{S\alpha}$. Activated adenylate cyclase eventually leads to an increase of intracellular cAMP level in most cellular systems (Holmgren, 1981). Modulation of cellular function by CT has been reported, including modification of cell growth by the toxin. For example, CT stimulates the growth of cultured human mammary epithelial cells (Taylor-Papadimitriou *et al.*, 1980), Swiss 3T3 cells (Rozengurt *et al.*, 1981), and epithelial cells from normal human bronchus (Lechner *et al.*, 1981), in the presence of serum or growth factors. In contrast, it has also been shown that CT inhibits TGF-β1-induced monolayer growth of fibroblast cells (How *et al.*, 1989) and the proliferation of hormone-dependent rat mammary cancer cells (Cho-Chung *et al.*, 1983) and human small-cell lung cancer cells (Viallet *et al.*, 1990). Although the mechanism(s) of CT-induced cellular events is not definitely known at present, it is believed that increased intracellular cAMP is a participating element. However, Viallet *et al.* have reported that elevation of cellular cAMP alone can not account for CT-induced growth inhibition of human small-cell lung cancer (Viallet *et al.*, 1990).

On the other hand, the physiological actions of cAMP, a second messenger in a variety of extracellular signals, have been studied widely and extensively using its analogues. Among these analogues, 8-Cl-cAMP has been found to bind selectively to the site 1 receptor of the type II regulatory subunit (Cho-Chung, 1990). This site-selective cAMP analogue inhibits the cell growth of human colon cancer cell (Ally *et al.*, 1988; Tagliaferri *et al.*, 1988), leukemia cells (Tortora *et al.*, 1988), lung cancer cells (Ally *et al.*, 1989), gastric cancer cells (Takanashi *et al.*, 1991), and Ki-*ras*-transformed rat fibroblasts (Tortora *et al.*, 1989).

Correspondence:
Received 19 May 1992; and in revised form 8 September 1992.

Previously, we have shown that the growth of human pancreatic cancer cells (MIA PaCa-2) is enhanced by transforming growth factor (TGF)-α and insulin-like growth factor (IGF)-I in an autocrine manner (Ohmura *et al.*, 1990). In the present study, we demonstrate that CT and 8-Cl-cAMP suppress pancreatic cancer cell growth, and the mechanism(s) of actions of these substances was also investigated, in relation to the above two growth factors and K-ras oncogene, where the point mutation is frequently observed in pancreatic carcinomas (Barbacid, 1987; Barbacid, 1990).

### Materials and methods

### *Reagents and cell culture*

CT, forskolin and TPA were purchased from Sigma Chemical Co. (St. Louis, MO). 8-Cl-cAMP was provided by ICN Pharmaceuticals (Costa Mesa, CA).

Human pancreatic carcinoma cells (MIA PaCa-2, Panc-1) were obtained from American Type Culture Collection (Rockville, MD) and Japanese Cancer Research Resources Bank (Tokyo, Japan), respectively. The cells were maintained by once-weekly passage in Dulbecco's modified essential medium (DMEM) containing 5% fetal calf serum (FCS, Filtron, Australia) at 37°C in 5% $CO_2$/95% humidified air.

### *Proliferation study*

MIA PaCa-2 cells (PC cells) and Panc-1 cells were seeded into 12-well plates (Costar, MA) at a density of 1.2–1.7 × $10^4$ cells/well. Cultured cells were used after growing for 2 days in 5% FCS/DMEM. The cells were washed with serum-free DMEM; the proliferation studies were then performed in DMEM supplemented with 5% FCS (5% FCS/DMEM) or 0.3% bovine serum albumin (BSA) (0.3% BSA/DMEM). The test materials, dissolved with 0.3% BSA/DMEM, were added at the beginning of the experiment. Media and materials were changed every 2 days. At the end of experiments (usually 4 days after adding the test materials unless otherwise stated), cells isolated by trypsinizing were counted using a Coulter Counter (Coulter Electronics Inc.).

The mean ± s.d. was calculated for each group and significance was determined by the Student's *t* test.

### *Binding studies*

Subconfluent cells cultured in 12-well plates were used for the binding studies. The cells were washed once with ice-cold 0.3% BSA/DMEM supplemented with 20 mmol $l^{-1}$ 4-(2-hydroxyethyl)-1-piperazine ethanesulfonic acid (pH; 7.4) and then incubated at 4°C for 24 h in the same medium with or without peptides. Labelled ligands (40,000–50,000 c.p.m./well) were added and incubated at 4°C for 20 h. After removing the medium, the cells were washed twice with ice-cold fresh medium and solubilised with 0.5 ml 1 N NaOH. Samples were transferred to tubes for radioactivity counts.

### *cAMP assay*

Subconfluent cultured cells were incubated for 24 h with or without CT in 0.3% BSA/DMEM containing 0.5 mmol $l^{-1}$ 3-isobutyl-1-methyl-xanthine. The cells and medium were then extracted with 10% TCA. The amount of cAMP in the supernatant was determined using cAMP RIA kits (New England Nuclear, Boston, MA).

### *Immunoblotting*

Subconfluent PC cells cultured in 5% FCS/DMEM were collected and suspended in 5 ml 10 m mol $l^{-1}$ Tris-HCl (pH; 7.4) containing 10% sucrose, 1 m mol $l^{-1}$ phenylmethylsulfonyl fluoride (PMSF) and 50 units $ml^{-1}$ aprotinin. Cells were homogenised with a Teflon glass homogeniser, sonicated for 2 min, and then centrifuged at 600 *g* for 10 min. Supernatants were centrifuged at 20,000 *g* for 40 min. The pellets were suspended in 0.5 ml 10 m mol $l^{-1}$ Tris-HCl (pH; 7.4) containing 0.15 mol $l^{-1}$ NaCl, 0.01 mol $l^{-1}$ $MaCl_2$, 0.5% Nonidet P-40, 1 m mol $l^{-1}$ PMSF, and 50 units $ml^{-1}$ aprotinin. The suspension was centrifuged at 20,000 *g* for 40 min at 4°C. Supernatants (300 μg protein/lane) were separated by 17% SDS-PAGE and transferred to Clear Blot Membrane-P (Atto, Tokyo, Japan). K-*ras* p21 (Oncogene Science, Inc. Manhasset, NY) was used for the control antigen. After blocking with 3% BSA, the membranes were incubated with monoclonal antibody to K-*ras* p21 (Oncogene Science, Inc.) or normal mouse IgG for 20 h at 4°C and then incubated with $^{125}$I-labelled protein A for 16 h at 4°C. They were exposed to an X-ray film (Fuji X-ray, Tokyo, Japan) at – 70°C for 3–4 days. The quantitative analysis was performed with Video densitometer (Nippon Bio Rad Lab. Tokyo, Japan).

### Results

### *Effect of CT on growth and cAMP production in PC or Panc-1 cells*

As shown in Figure 1a, CT suppressed PC cell growth in the absence (0.3% BSA) or presence of 5% FCS. Half the maximum suppressive dose ($IC_{50}$) of CT was between 10–100 pg $ml^{-1}$. The inhibitory effect of the toxin appears to be reversible because PC cells grew again after the elimination of CT in the medium (Figure 2). In contrast, CT was far less effective on the cell growth of Panc-1 cells, another pancreatic cancer cell line (Figure 1b). It is generally assumed that the action of CT is attributed in part to cAMP stimulated by the toxin. To address this possibility, we observed the cAMP production of these two kinds of cells exposed to CT. As shown in Figure 3, at low levels of 1 pg $ml^{-1}$ CT was able to stimulate cAMP production in PC cells. At a concentration of 100 pg $ml^{-1}$ of the toxin, the production of cAMP was enhanced 320-fold (control; 16.4 ± 0.8 nmol $l^{-1}$, CT 100 pg $ml^{-1}$; 5230 ± 456 nmol $l^{-1}$).

**Figure 1a,b** Effect of CT on pancreatic cell growth (**a** MIA PaCa-2 cells (PC cells), **b** Panc-1 cells). Cells were cultured in 5% FCS/DMEM for 2 days. After removing the medium, cells were incubated with CT in 5% FCS/DMEM or 0.3% BSA/DMEM for 4 days. Medium and the test material were changed every 2 days. Values are means + s.d. (bars) of three determinations. (*$P < 0.01$).

**Figure 2** Time-course study of CT-induced suppression of PC (MIA PaCa-2) cell growth. Cells cultured with 5% FCS/DMEM were exposed to CT for 4 days. CT was then eliminated from the medium. Medium and test materials were changed every 2 days. (O—O; control, ●—●; CT 10 pg $ml^{-1}$, □—□; 100 pg $ml^{-1}$). Values are means ± s.d. (bars) of three determinations. Points without bars include s.d. in the symbols.

**Figure 3** Effect of CT on cAMP production of PC cells (□—□) and Panc-1 cells (●—●). For details see 'Materials and methods'. Values are means ± s.d. (bars) of three determinations.

Panc-1 cells, however, were less sensitive to CT in cAMP production (control; $14.3 \pm 2.2$ nmol $l^{-1}$, CT 100 pg $ml^{-1}$; $1408 \pm 89$ nmol $l^{-1}$).

*Effects of cAMP analogues and forskolin on cell growth of PC cells*

To clarify the mechanism of the growth-inhibitory effect of CT, the growth responses to cAMP analogues were investigated. 8-Cl-cAMP markedly suppressed the proliferation of PC cells cultured in 5% FCS/DMEM to 14% of control values ($IC_{50}$; 10–50 µmol $l^{-1}$; Figure 4a). This effect was also observed in PC cells cultured in serum-free medium (0.3% BSA/DMEM). Similarly, 8-Br-cAMP, another cAMP analogue, also had an inhibitory action ($IC_{50}$; 1 mmol $l^{-1}$), although it was less potent than 8-Cl-cAMP (data not shown). Furthermore, forskolin, which elevates the intracellular cAMP level, was able to suppress PC cell growth in a dose-responsive manner (Figure 4b). However, 8-Cl-cAMP was far less effective in Panc-1 cell growth (data not shown).

**Figure 4a,b** Effects of 8-Cl-cAMP **a**, and forskolin **b**, on PC cell growth. Experiments were performed as described in Figure 1. Cells were exposed to the materials for 4 days, the cell number was then counted. Values are means + s.d. (bars) of three determinations. (*; $P < 0.01$ *vs* control).

*Effect of CT and 8-Cl-cAMP on EGF or IGF-I binding to PC cells*

We have reported that TGF-α and IGF-I act as growth stimulators of PC cells in an autocrine fashion. To investigate the roles of these two growth factors in the inhibitory effect of CT or 8-Cl-cAMP, we measured the binding of $^{125}$I-EGF and $^{125}$I-IGF-I to cells treated with CT or 8-Cl-cAMP. However, they did not have substantial effects on the bindings of the growth factors to PC cells exposed to CT or 8-Cl-cAMP. A similar phenomenon was noted in Panc-1 cells exposed to 8-Cl-cAMP (data not shown).

*Effect of CT and 8-Cl-cAMP on levels of K-*ras *p21 in PC cells*

It has been suggested that mutated ras p21, a product of the *ras* oncogene, is responsible for the development of many types of cancer. In particular, the point mutation of K-*ras* oncogene is frequently (80%–90%) observed in human pancreatic cancer. This prompted us to examine whether K-*ras* p21 is modulated or not in PC cells treated by CT or 8-Cl-cAMP. As shown in Figure 5a and b, the level of K-*ras*

**Figure 5a,b** Effects of 8-Cl-cAMP and CT on K-*ras* p21 protein levels in PC cells analysed by Western blotting **a**. Quantitative analysis by a densitometer was shown in **b**. Cells were exposed to 8-Cl-cAMP (200 µmol $l^{-1}$) or CT (1 ng $ml^{-1}$) for 24 h and 72 h. Protein (300 µg/lane) was used for the analysis. The data are representative of three separate experiments. For details, see 'Materials and methods'.

**Figure 6** Interaction between CT, 8-Cl-cAMP, and TPA in PC cell growth. Experiments were done as described in Figure 1. The indicated concentrations of the materials were added to the medium simultaneously. Cells were counted after 4 days. (*; $P<0.01$). Values are means + s.d. (bars) of three determinations.

p21 was increased in the cells exposed to CT or 8-Cl-cAMP for 24 h. Even at 72 h, these substances did not suppress the protein levels. When normal IgG was used instead of anti-K-ras antibody, no bands were observed (data not shown).

*Combined effects of CT, 8-Cl-cAMP, and phorbol ester on PC cell growth*

We have previously shown that phorbol ester, an activator of protein kinase-C, also has the ability to suppress the proliferation of PC cells (Ohmura *et al.*, 1990). To investigate the additive or synergistic effect of CT, 8-Cl-cAMP, and phorbol ester (TPA), maximum suppressive doses of two of these substances were added to the medium simultaneously, and the interactions between the substances were examined. Figure 6 shows the result, demonstrating that PC cell growth was most strongly inhibited by 8-Cl-cAMP, which was further enhanced by TPA, but not CT. Similarly, CT-induced growth inhibition was further suppressed by TPA.

### Discussion

We have demonstrated that CT and 8-Cl-cAMP have the ability to inhibit the proliferation of PC cells. A low dose of CT (0.1 pg ml $l^{-1}$) was effective and $IC_{50}$ was between 10 pg $ml^{-1}$ and 100 pg $ml^{-1}$; Panc-1 cells, however, were far less sensitive to CT. Recently, Viallet *et al.* have shown that CT inhibits the growth of small-cell lung cancers with an $IC_{50}$ of 27–242 ng $ml^{-1}$ (Viallet *et al.*, 1990), which is higher than that observed in PC cells. Such a difference between the cell types in the sensitivity of the cells to CT may be explained partly by the difference of the expression of cellular binding sites for CT, i.e. $G_M$ ganglioside (Viallet *et al.*, 1990). Although the precise mechanism of CT-induced growth inhibition is not clear at present, it is generally assumed that increases in cellular cAMP may be involved in the growth inhibition by CT. However, the existence of a different mechanism is suggested in the action of CT. Proliferation in such conditions as small-cell lung cancer, which is sensitive to CT, but not inhibited by cAMP analogues and forskolin, implies that CT acts through a cAMP-independent mechanism (Viallet *et al.*, 1990). In the present study, cellular cAMP production of PC cells was stimulated by CT (Figure 3). Forksolin, which increases cellular cAMP, was also effective in suppressing PC cell growth. Further, the addition of 8-Cl-cAMP (Figure 4a, $IC_{50}$; 10–50 $\mu$mol $l^{-1}$) or 8-Br-cAMP ($IC_{50}$; 1 mmol $l^{-1}$) inhibited the growth rate of PC cells. Such different $IC_{50}$ values may imply that the growth inhibition is not due to high concentration of nucleotides. Above observations suggest that the suppressive effect of CT is mediated, at least partly, through the increase of intracellular cAMP enhanced by the toxin.

Previously, we have reported that IGF-I and TGF-α act as autocrine growth stimulators in PC cell growth (Ohmura *et al.*, 1990). This prompted us to determine whether the receptors for these growth factors are modified by CT or 8-Cl-cAMP. The results showed that CT and 8-Cl-cAMP had no substantial effects on the bindings of these peptide to PC cells, suggesting that the growth inhibition by CT or 8-Cl-cAMP does not occur at the binding sites of IGF-I or TGF-α. With regard to EGF/TGF-α receptor, it has been reported that 8-Cl-cAMP does not modify EGF-receptor mRNA in human gastric carcinoma cell lines (Takanashi *et al.*, 1991), which is consistent with our own observations.

The *ras* oncogenes, which are associated with many types of tumours, acquire their transforming property either by single point mutations or over-expression of the normal *ras* p21 (Barbacid, 1987; Barbacid, 1990). In particular, activating mutations of the K-*ras* gene at codons 12, 13, or 61, have been frequently (90%) observed in human pancreatic cancers, and it is a higher rate than those found in other human tumours. Although the functional roles of activated K-*ras* oncogenes are not clear, it has been speculated that the oncogene products play important roles in signal transductions because of their structural and biochemical similarities to G protein (Barbacid, 1987; Barbacid, 1990). When antibodies specific for amino acid 12 of v-Ki-*ras* protein are microinjected into cells transformed by this protein, the cells transiently reverse to a normal phenotype (Feramisco *et al.*, 1985). According to a recent report, when the expression of the mutated *ras* p21 was down-regulated by anti-sense RNA, the growth of human cancer cells was inhibited, suggesting that the mutated p21 protein contributes to the faster growth rate of these cells (Mukhopadhyay *et al.*, 1991). To determine whether CT or 8-Cl-cAMP modifies K-*ras* p21 production, the protein of PC cells, in which the point mutation was also observed at codon 12 (data not shown), was analysed by Western blotting. Our results, however, demonstrate that CT and 8-Cl-cAMP increased K-*ras* p21 at 24 h. The relationship between 8-Cl-cAMP and *ras* p21 has been shown by other investigators in different cell lines. In human lung carcinoma, the expression of N-*ras* mRNA is increased by 8-Cl-cAMP at 1 h and 6 h; however, it is decreased at 7 days (Ally *et al.*, 1989). Also, H-*ras* mRNA expression was increased in gastric cancer cells exposed to 8-Cl-cAMP for 24 h and 48 h (Takanashi *et al.*, 1991). Our results are consistent with these observations. Chesa *et al.* have reported that terminally differentiated cells showed stronger reactivity with antibody against *ras* p21 than did rapidly proliferating cells (Chesa *et al.*, 1987). Thus, it may be likely that there is a relationship between growth inhibition by CT or 8-Cl-cAMP and increased K-*ras* p21. By contrast, there are reports which indicate that 8-Cl-cAMP reduces the p21 level: 8-Cl-cAMP treatment for 3 days caused reduction of p21 in breast cancer cell lines (Taglioferri *et al.*, 1988); 8-Cl-cAMP treatment also decreased the levels of p21 protein in K-*ras*-transformed rat fibroblasts (Tortora *et al.*, 1989). This discrepancy may be explained partly by differences in the cells used and/or antibody, i.e. we employed specific anti-K-*ras* p21 antibody, which did not react with either H- or N-*ras* p21, whereas some investigators used an antibody that reacts with whole p21 protein. However, the exact roles of K-*ras* p21 on PC cell growth remains to be elucidated.

As already indicated, activators of protein kinase C, such as TPA, decrease the affinity or number of EGF receptors (Shoyab *et al.*, 1979; Lee & Weinstein, 1978) and stimulate the phosphorylation of the EGF receptor to inhibit its functions (Iwashita & Fox, 1984; Cochet *et al.*, 1984). We have also shown that TPA markedly reduces the affinity of EGF/TGF-α receptor in PC cells and possibly causes growth inhibition by TPA (Ohmura *et al.*, 1990). Our present study revealed that CT and 8-Cl-cAMP did not reduce the binding of EGF, suggesting that these substances and TPA act through different mechanisms. This prompted us to examine the interaction between CT or 8-Cl-cAMP and TPA. As shown in Figure 6, suppressed PC cell growth by almost maximum dose of 8-Cl-cAMP was further inhibited by TPA, but not by CT. Similarly CT-induced growth inhibition was further suppressed by TPA. These data support the view that the inhibitory action of CT or 8-Cl-cAMP is different from that of TPA, and a combination of these substances strengthens the anti-proliferative effect of each.

In conclusion, we have shown that CT and 8-Cl-cAMP suppress PC cell growth; the mechanism is presumably different from that of TPA. The finding that the action of CT or 8-Cl-cAMP is enhanced by TPA raises the possibility that a combination of these substances may be useful for the growth control of malignant cells.

We wish to thank Mariko Arai and Yasuko Sato for their expert assistance. The present work was supported by grants from the Ministry of Education, Ministry of Health and Welfare of Japan, and the Foundation for Growth Science in Japan.

### References

ALLY, S., TORTORA, G., CLAIR, T., GRIECO, D., MERLO, G., KATSAROS, D., OGREID, D., DOSKELAND, S.O., JAHNSEN, T. & CHO-CHUNG, Y.S. (1988). Selective modulation of protein kinase isozymes by the site-selective analog 8-chloroadenosine 3′,5′-cyclic monophosphate provides a biological means for control of human colon cancer cell growth. *Proc. Natl Acad. Sci. USA*, **85**, 6319–6322.

ALLY, S., CLAIR, T., KATSAROS, D., TORTORA, G., YOKOZAKI, H., FINCH, R.A., AVERY, T.L. & CHO-CHUNG, Y.S. (1989). Inhibition of growth and modulation of gene expression in human lung carcinoma in athymic mice by site-selective 8-Cl-cyclic adenosine monophosphate. *Cancer Res.*, **49**, 5650–5655.

BARBACID, M. (1987). ras Genes. *Ann. Rev. Biochem.*, **56**, 779–827.

BARBACID, M. (1990). ras Oncogenes: their role in neoplasia. *Euro. J. Clin. Invest.*, **20**, 225–235.

CHESA, P., RETTIG, W.J., MELAMED, M.R., OLD, L.J. & NIMAN, H.L. (1987). Expression of $p21^{ras}$ in normal and malignant human tissues: lack of association with proliferation and malignancy. *Proc. Natl Acad. Sci. USA*, **84**, 3234–3238.

CHO-CHUNG, Y.S., CLAIR, T., SHEPHEARD, C. & BERGHOFFER, B. (1983). Arrest of hormone-dependent mammary cancer growth *in vivo* and *in vitro* by cholera toxin. *Cancer Res.*, **43**, 1473–1476.

CHO-CHUNG, Y.S. (1990). Role of cyclic AMP receptor proteins in growth, differentiation, and suppression of malignancy: new approaches to therapy. *Cancer Res.*, **50**, 7093–7100.

COCHET, C., GILL, G.N., MEISENHELDER, J., COOPER, J.A. & HUNTER, T. (1984). C-kinase phosphorylates the epidermal growth factor receptor and reduces its epidermal growth factor-stimulated tyrosine protein kinase activity. *J. Biol. Chem.*, **259**, 2553–2558.

FERAMISCO, J.R., CLARK, R., WONG, G., ARNHEIM, N., MILLEY, R. & MCCORMICK, F. (1985). Transient reversion of ras oncogene-induced cell transformation by antibodies specific for amino acid 12 of ras protein. *Nature*, **314**, 639–642.

HOLMGREN, J. (1981). Action of cholera toxin and the prevention and treatment of cholera. *Nature*, **292**, 413–417.

HOW, P.H., BASCOM, C.C., CUNNINGHAM, M.R. & LEOF, E.B. (1989). Regulation of transforming growth factor β1 action by multiple transducing pathways: evidence for both G protein-dependent and -independent signaling. *Cancer Res.*, **49**, 6024–6031.

IWASHITA, S. & FOX, F. (1984). Epidermal growth factor and potent phorbol tumor promoters induce epidermal growth factor receptor phosphorylation in a similar but distinctively different manner in human epidermoid carcinoma A431 cells. *J. Biol. Chem.*, **259**, 2559–2567.

LECHNER, J.F., HAUGEN, A., AUTRUP, H., MCCLENDON, I.A., TRUMP, B.F. & HARRIS, C.C. (1981). Clonal growth of epithelial cells from normal adult human bronchus. *Cancer Res.*, **41**, 2294–2304.

LEE, L.S. & WEINSTEIN, I.B. (1978). Tumor-promoting phorbol esters inhibit binding of epidermal growth factor to cellular receptors. *Science*, **202**, 313–315.

MUKHOPADHYAY, T., TAINSKY, M., CAVENDER, A.C. & ROTH, J.A. (1991). Specific inhibition of K-ras expression and tumorgenicity of lung cancer cells by antisense RNA. *Cancer Res.*, **51**, 1744–1748.

OHMURA, E., OKADA, M., ONODA, N., KAMIYA, Y., MURAKAMI, H., TSUSHIMA, T. & SHIZUME, K. (1990). Insulin-like growth factor I and transforming growth factor α as autocrine growth factors in human pancreatic cancer cell growth. *Cancer Res.*, **50**, 103–107.

ROZENGURT, E., LEGG, A., STRANG, G. & COURTENAY-LUCK, N. (1981). Cyclic AMP: a mitogenic signal for Swiss 3T3 cells. *Proc. Natl Acad. Sci. USA*, **78**, 4392–4396.

SHOYAB, M., DE LARCO, J.E. & TODARO, G.J. (1979). Biologically active phorbol esters specifically alter affinity of epidermal growth factor membrane receptors. *Nature*, **279**, 387–391.

TAGLIAFERRI, P., KATSAROS, D., CLAIR, T., ALLY, S., TORTORA, G., NECKERS, L., RUBALCAVA, B., PARANDOOSH, Z., CHANG, Y., REVANKAR, G.R., CRABTREE, G.W., ROBINS, R.K. & CHO-CHUNG, Y.S. (1988). Synergistic inhibition of growth of breast and colon human cancer cell lines by site-selective cyclic AMP analogues. *Cancer Res.*, **48**, 1642–1650.

TAKANASHI, A., YASUI, W., YOSHIDA, K., YOKOZAKI, H., SAITO, D., ABE, K., URAKAMI, K., MIKI, K. & TAHARA, E. (1991). Inhibitory effect of 8-chloro-cyclic adenosine 3′, 5′-monophosphate on cell growth of gastric carcinoma cell lines. *Jpn. J. Cancer Res.*, **82**, 325–331.

TAYLOR-PAPADIMITRIOU, J., PURKIS, P. & FENTIMAN, I.S. (1980). Cholera toxin and analogues of cyclic AMP stimulate growth of cultured human mammary epithelial cells. *J. Cell. Physiol.*, **102**, 317–321.

TORTORA, G., TAGLIAFERRI, P., CLAIR, T., COLAMONICI, O., NECKERS, L.M., ROBINS, R.K. & CHO-CHUNG, Y.S. (1988). Site-selective cAMP analogs at micromolar concentrations induce growth arrest and differentiation of acute promyelocytic, chronic myelocytic, and acute lymphocytic human leukemia cell lines. *Blood*, **71**, 230–233.

TORTORA, G., CIARDIELLO, F., ALLY, S., CLAIR, T., SALOMON, D.S. & CHO-CHUNG, Y.S. (1989). Site-selective 8-chloroadenosine 3′, 5′-cyclic monophosphate inhibits transformation and transforming growth factor α production in Ki-*ras*-transformed rat fibroblasts. *FEBS Lett.*, **242**, 363–367.

VIALLET, J., SHARONI, Y., FRUCHT, H., JENSEN, R.J., MINNA, J.D. & SAUSVILLE, E.A. (1990). Cholera toxin inhibits signal transduction by several mitogens and the *in vitro* growth of human small-cell lung cancer. *J. Clin. Invest.*, **86**, 1904–1912.


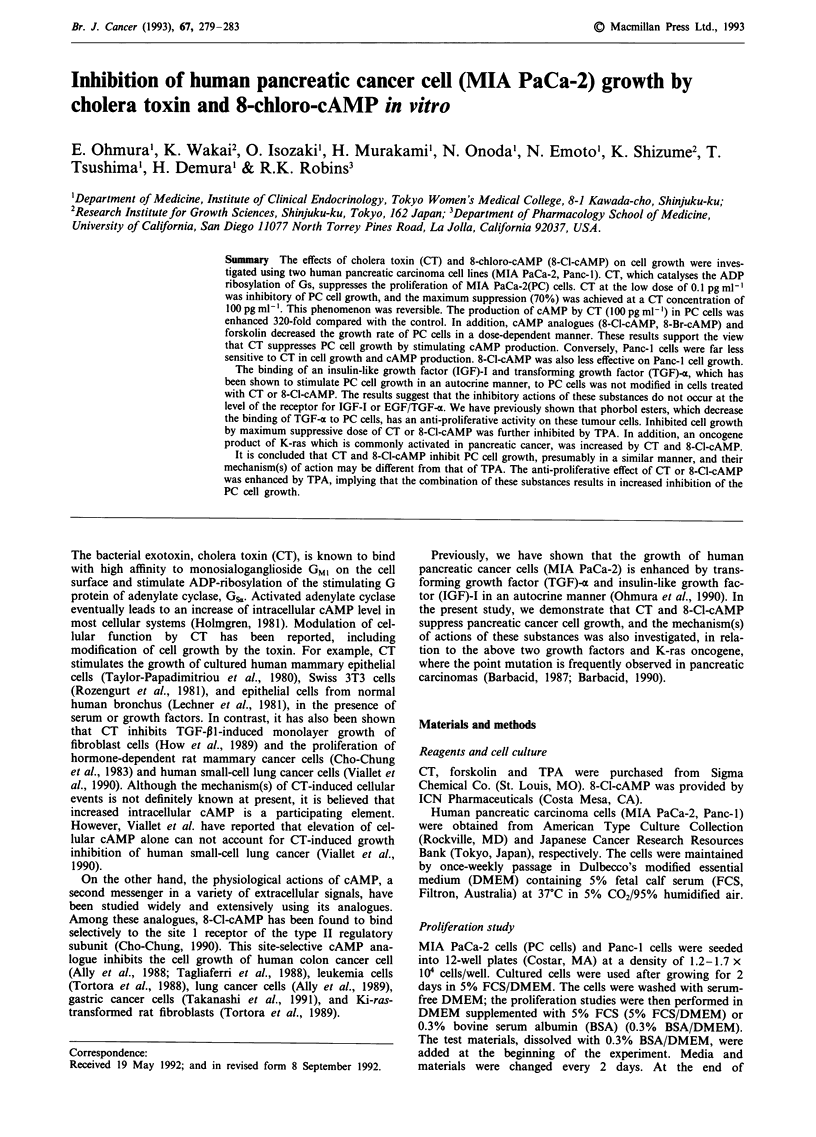

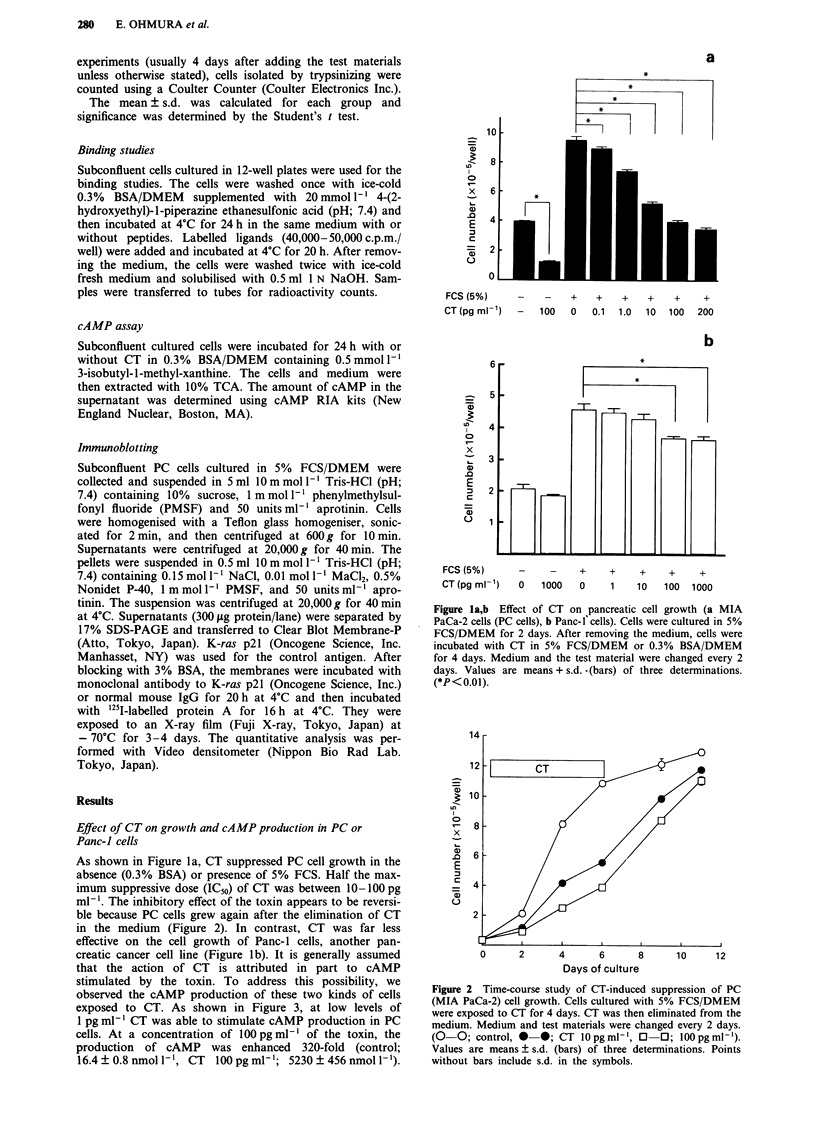

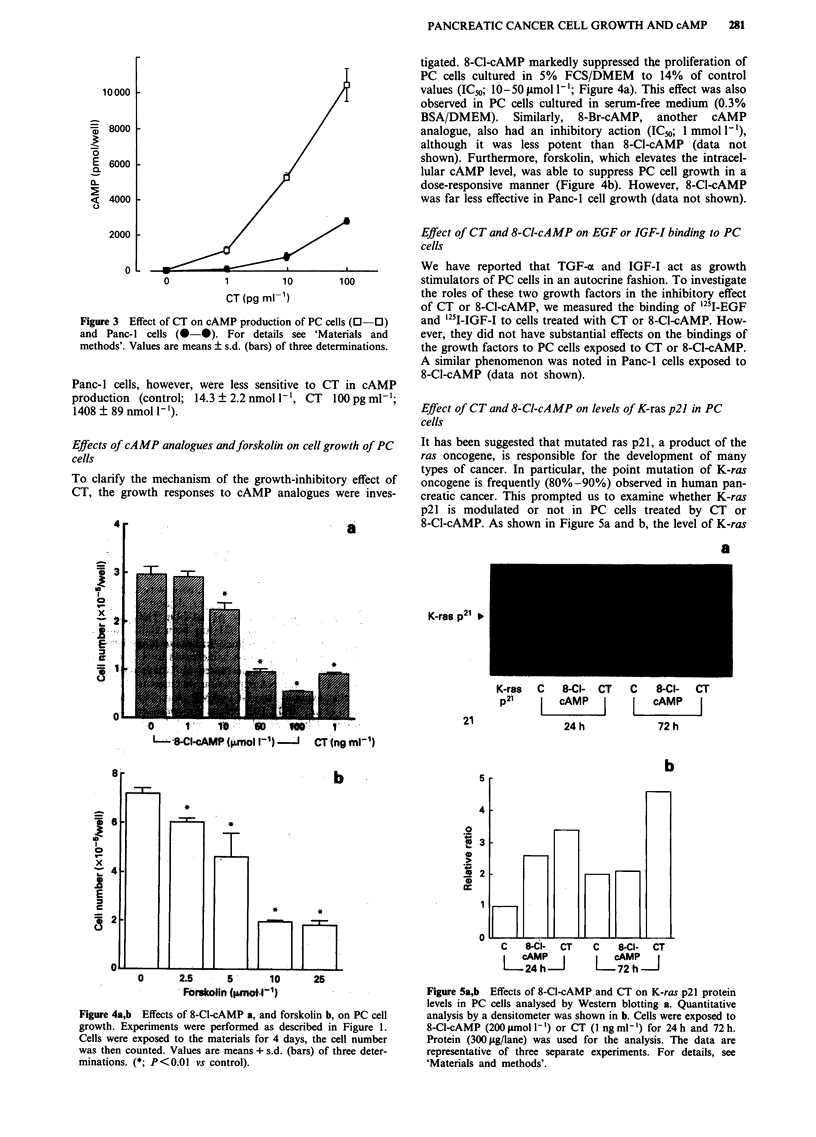

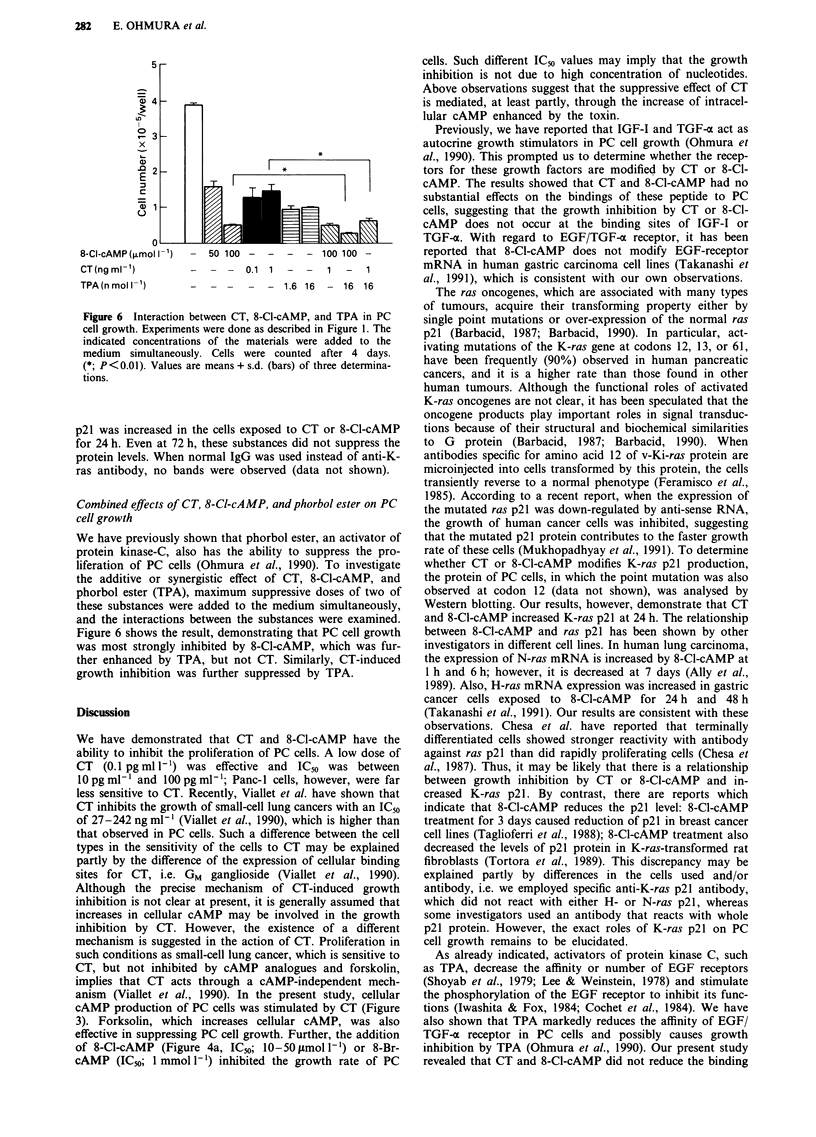

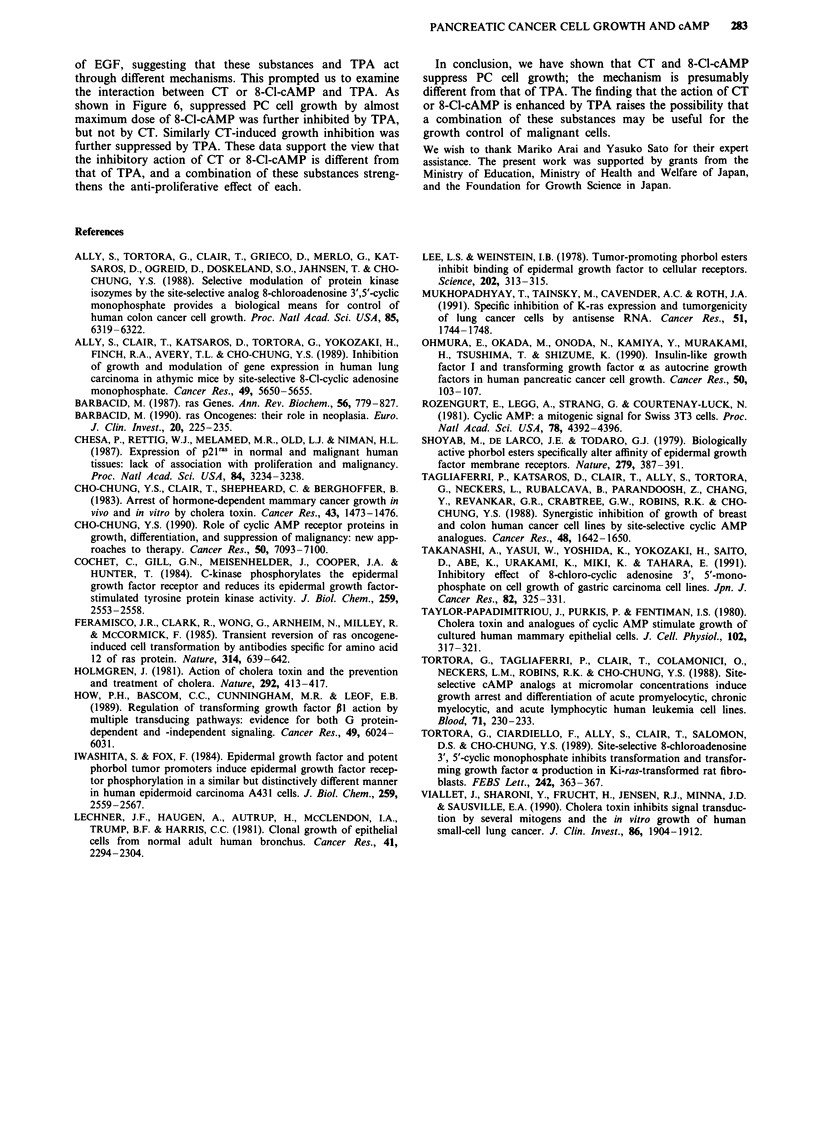


## References

[OCR_00689] Ally S., Clair T., Katsaros D., Tortora G., Yokozaki H., Finch R. A., Avery T. L., Cho-Chung Y. S. (1989). Inhibition of growth and modulation of gene expression in human lung carcinoma in athymic mice by site-selective 8-Cl-cyclic adenosine monophosphate.. Cancer Res.

[OCR_00683] Ally S., Tortora G., Clair T., Grieco D., Merlo G., Katsaros D., Ogreid D., Døskeland S. O., Jahnsen T., Cho-Chung Y. S. (1988). Selective modulation of protein kinase isozymes by the site-selective analog 8-chloroadenosine 3',5'-cyclic monophosphate provides a biological means for control of human colon cancer cell growth.. Proc Natl Acad Sci U S A.

[OCR_00696] Barbacid M. (1987). ras genes.. Annu Rev Biochem.

[OCR_00697] Barbacid M. (1990). ras oncogenes: their role in neoplasia.. Eur J Clin Invest.

[OCR_00701] Chesa P. G., Rettig W. J., Melamed M. R., Old L. J., Niman H. L. (1987). Expression of p21ras in normal and malignant human tissues: lack of association with proliferation and malignancy.. Proc Natl Acad Sci U S A.

[OCR_00707] Cho-Chung Y. S., Clair T., Shepheard C., Berghoffer B. (1983). Arrest of hormone-dependent mammary cancer growth in vivo and in vitro by cholera toxin.. Cancer Res.

[OCR_00711] Cho-Chung Y. S. (1990). Role of cyclic AMP receptor proteins in growth, differentiation, and suppression of malignancy: new approaches to therapy.. Cancer Res.

[OCR_00716] Cochet C., Gill G. N., Meisenhelder J., Cooper J. A., Hunter T. (1984). C-kinase phosphorylates the epidermal growth factor receptor and reduces its epidermal growth factor-stimulated tyrosine protein kinase activity.. J Biol Chem.

[OCR_00723] Feramisco J. R., Clark R., Wong G., Arnheim N., Milley R., McCormick F. (1985). Transient reversion of ras oncogene-induced cell transformation by antibodies specific for amino acid 12 of ras protein.. Nature.

[OCR_00729] Holmgren J. (1981). Actions of cholera toxin and the prevention and treatment of cholera.. Nature.

[OCR_00733] Howe P. H., Bascom C. C., Cunningham M. R., Leof E. B. (1989). Regulation of transforming growth factor beta 1 action by multiple transducing pathways: evidence for both G protein-dependent and -independent signaling.. Cancer Res.

[OCR_00740] Iwashita S., Fox C. F. (1984). Epidermal growth factor and potent phorbol tumor promoters induce epidermal growth factor receptor phosphorylation in a similar but distinctively different manner in human epidermoid carcinoma A431 cells.. J Biol Chem.

[OCR_00747] Lechner J. F., Haugen A., Autrup H., McClendon I. A., Trump B. F., Harris C. C. (1981). Clonal growth of epithelial cells from normal adult human bronchus.. Cancer Res.

[OCR_00753] Lee L. S., Weinstein I. B. (1978). Tumor-promoting phorbol esters inhibit binding of epidermal growth factor to cellular receptors.. Science.

[OCR_00758] Mukhopadhyay T., Tainsky M., Cavender A. C., Roth J. A. (1991). Specific inhibition of K-ras expression and tumorigenicity of lung cancer cells by antisense RNA.. Cancer Res.

[OCR_00764] Ohmura E., Okada M., Onoda N., Kamiya Y., Murakami H., Tsushima T., Shizume K. (1990). Insulin-like growth factor I and transforming growth factor alpha as autocrine growth factors in human pancreatic cancer cell growth.. Cancer Res.

[OCR_00771] Rozengurt E., Legg A., Strang G., Courtenay-Luck N. (1981). Cyclic AMP: a mitogenic signal for Swiss 3T3 cells.. Proc Natl Acad Sci U S A.

[OCR_00776] Shoyab M., De Larco J. E., Todaro G. J. (1979). Biologically active phorbol esters specifically alter affinity of epidermal growth factor membrane receptors.. Nature.

[OCR_00785] Tagliaferri P., Katsaros D., Clair T., Ally S., Tortora G., Neckers L., Rubalcava B., Parandoosh Z., Chang Y. A., Revankar G. R. (1988). Synergistic inhibition of growth of breast and colon human cancer cell lines by site-selective cyclic AMP analogues.. Cancer Res.

[OCR_00789] Takanashi A., Yasui W., Yoshida K., Yokozaki H., Saito D., Abe K., Urakami K., Miki K., Tahara E. (1991). Inhibitory effect of 8-chloro-cyclic adenosine 3',5'-monophosphate on cell growth of gastric carcinoma cell lines.. Jpn J Cancer Res.

[OCR_00796] Taylor-Papadimitriou J., Purkis P., Fentiman I. S. (1980). Cholera toxin and analogues of cyclic AMP stimulate the growth of cultured human mammary epithelial cells.. J Cell Physiol.

[OCR_00810] Tortora G., Ciardiello F., Ally S., Clair T., Salomon D. S., Cho-Chung Y. S. (1989). Site-selective 8-chloroadenosine 3',5'-cyclic monophosphate inhibits transformation and transforming growth factor alpha production in Ki-ras-transformed rat fibroblasts.. FEBS Lett.

[OCR_00802] Tortora G., Tagliaferri P., Clair T., Colamonici O., Neckers L. M., Robins R. K., Cho-Chung Y. S. (1988). Site-selective cAMP analogs at micromolar concentrations induce growth arrest and differentiation of acute promyelocytic, chronic myelocytic, and acute lymphocytic human leukemia cell lines.. Blood.

[OCR_00817] Viallet J., Sharoni Y., Frucht H., Jensen R. T., Minna J. D., Sausville E. A. (1990). Cholera toxin inhibits signal transduction by several mitogens and the in vitro growth of human small-cell lung cancer.. J Clin Invest.

